# The cyclic-di-GMP signaling pathway in the Lyme disease spirochete, *Borrelia burgdorferi*

**DOI:** 10.3389/fcimb.2014.00056

**Published:** 2014-05-01

**Authors:** Elizabeth A. Novak, Syed Z. Sultan, Md. A. Motaleb

**Affiliations:** Department of Microbiology and Immunology, East Carolina University Brody School of MedicineGreenville, NC, USA

**Keywords:** c-di-GMP, *Borrelia burgdorferi*, Lyme disease, motility, chemotaxis, virulence

## Abstract

In nature, the Lyme disease spirochete *Borrelia burgdorferi* cycles between the unrelated environments of the *Ixodes* tick vector and mammalian host. In order to survive transmission between hosts, *B. burgdorferi* must be able to not only detect changes in its environment, but also rapidly and appropriately respond to these changes. One manner in which this obligate parasite regulates and adapts to its changing environment is through cyclic-di-GMP (c-di-GMP) signaling. c-di-GMP has been shown to be instrumental in orchestrating the adaptation of *B. burgdorferi* to the tick environment. *B. burgdorferi* possesses only one set of c-di-GMP-metabolizing genes (one diguanylate cyclase and two distinct phosphodiesterases) and one c-di-GMP-binding PilZ-domain protein designated as PlzA. While studies in the realm of c-di-GMP signaling in *B. burgdorferi* have exploded in the last few years, there are still many more questions than answers. Elucidation of the importance of c-di-GMP signaling to *B. burgdorferi* may lead to the identification of mechanisms that are critical for the survival of *B. burgdorferi* in the tick phase of the enzootic cycle as well as potentially delineate a role (if any) c-di-GMP may play in the transmission and virulence of *B. burgdorferi* during the enzootic cycle, thereby enabling the development of effective drugs for the prevention and/or treatment of Lyme disease.

## Introduction

*Borrelia burgdorferi* is the causative agent of Lyme disease or Lyme borreliosis—the most common arthropod-borne disease in the United States and Europe (Adams et al., 2012, 2013). Although the total number of reported cases of Lyme disease each year in the United States averages at about 30,000 (Adams et al., 2013), the Centers for Disease Control and Prevention (CDC) estimates that the actual number of people diagnosed with Lyme disease each year is approximately 300,000 (Kuehn, 2013). However, while this disease has been reported in every state, it is substantially concentrated in the states of the upper Midwest and Northeast (Adams et al., 2013). The concentration of Lyme disease in these areas is directly related to the large population of the white-footed mouse (*Peromyscus leucopus*), which is thought to be the main reservoir of the Lyme disease spirochete (Levine et al., 1985; Mather et al., 1989; Ostfeld, 1997; Schmidt and Ostfeld, 2001; Tsao, 2009). Despite tremendous amounts of research and many promising results, there is no current vaccine available which protects humans against Lyme disease. Prevention—reducing exposure to ticks as well as to areas where ticks and their reservoir hosts may congregate—is currently the best defense against Lyme disease (Barrett and Portsmouth, 2013). However, the incidence and geographical distribution of Lyme disease is increasing, and is predicted to continue, in conjunction with the increasing overlap between humans, ticks, and their reservoir hosts (Ostfeld, 1997). Additionally, the persistent and debilitating nature of the disease leads to approximately two billion dollars in direct medical expenses and lost productivity each year in the United States (Maes et al., 1998; Zhang et al., 2006). Thus, these circumstances clearly warrant a basic understanding of the disease mechanism, which could lead to the development of a vaccine for the treatment (and potentially prevention) of Lyme disease.

Cyclic di-GMP (c-di-GMP) [bis(3′,5′)-cyclic diguanylic acid] was initially identified as a nucleotide-based, allosteric activator of cellulose synthase activity in *Gluconacetobacter xylinus* (formerly *Acetobacter xylinum*) (Ross et al., 1987). Since then, c-di-GMP has evolved as a broadly conserved second messenger unique to bacteria, which operates as a global regulatory molecule capable of altering a plethora of biological processes, including, but not limited to exopolysaccharide matrix components (Jenal and Malone, 2006; Hengge, 2009), virulence of plant and animal pathogens (Cotter and Stibitz, 2007; Tamayo et al., 2007, 2008; Chatterjee et al., 2008; McCarthy et al., 2008; Hammer and Bassler, 2009; Lai et al., 2009), motility and sessility (Wolfe and Visick, 2008; Armitage and Berry, 2010; Boehm et al., 2010; Paul et al., 2010), regulated proteolysis and cell cycle progression (Duerig et al., 2009), photosynthesis (Thomas et al., 2004), heavy metal resistance (Brown et al., 1986; Römling et al., 2005), and phage resistance (Chae and Yoo, 1986; Römling et al., 2005). These physiological functions regulated by c-di-GMP are controlled at the transcriptional, translational, and posttranslational levels (Ryjenkov et al., 2006; Weber et al., 2006; Lee et al., 2007; Merighi et al., 2007; Monds et al., 2007; Hickman and Harwood, 2008; Pesavento et al., 2008; Sudarsan et al., 2008; Duerig et al., 2009; Boehm et al., 2010; Fang and Gomelsky, 2010; Paul et al., 2010). This is in contrast to canonical two-component signal transduction systems, where a signal is detected by a sensor kinase, which phosphorylates its cognate response regulator, leading to the modulation of a limited number of genes (Albright et al., 1989). There are at least four components needed for functional activity of a c-di-GMP “control module”: a diguanylate cyclase (DGC) that synthesizes c-di-GMP; a phosphodiesterase (PDE) which hydrolyzes c-di-GMP; a receptor component that senses c-di-GMP by directly binding to it; and a target that directly contacts and is controlled by the effector, resulting in an output that is determined by the interplay of all of these components.

**Figure 1:**
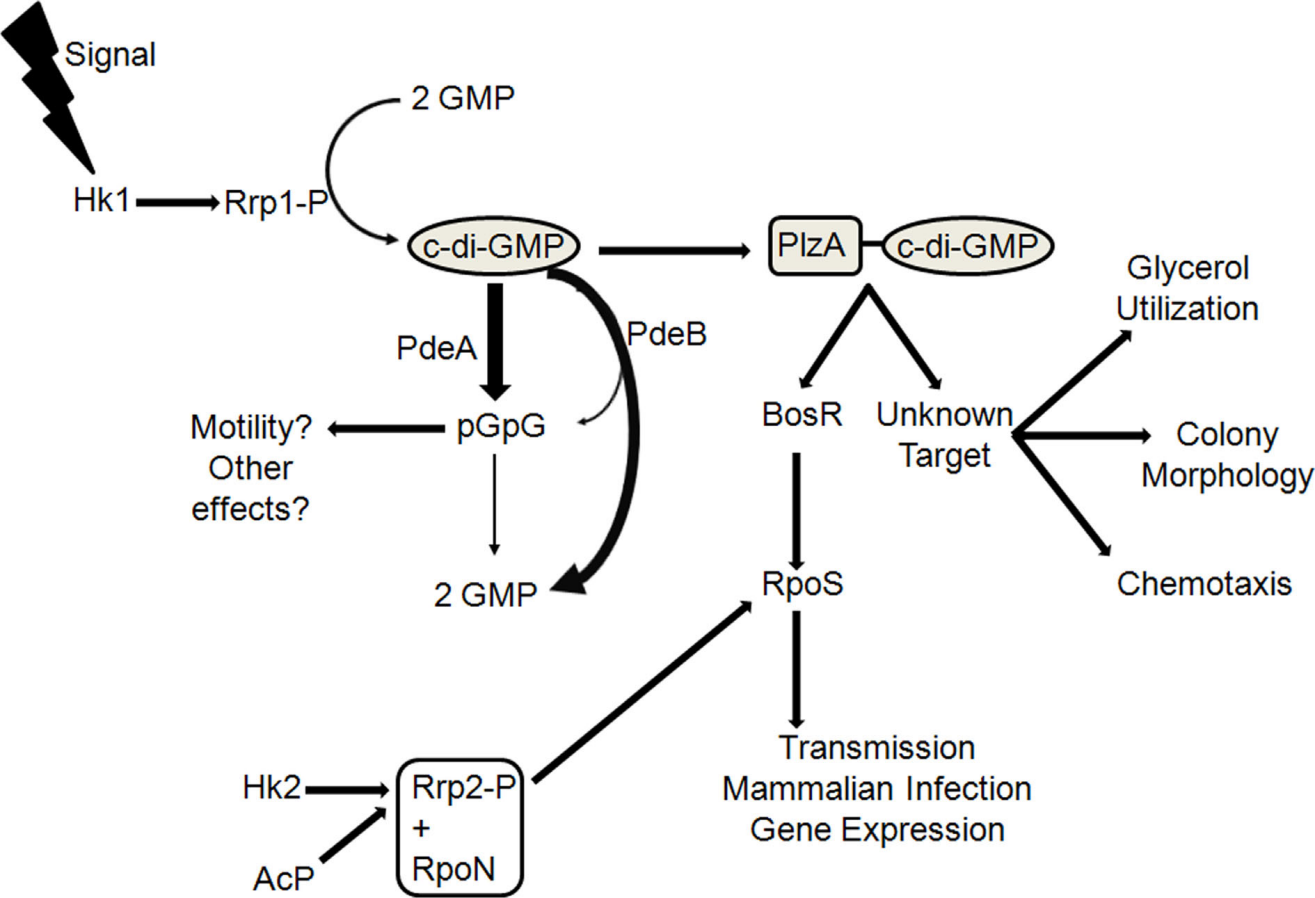
**A simplified working model of the c-di-GMP signaling pathway in *B. burgdorferi***. Hk1 and Rrp1 are present in the same operon and are thought to comprise a TCS. The diguanylate cyclase activity of Rrp1 is dependent upon its phosphorylation state, which presumably occurs in the cell by its cognate sensor kinase, Hk1. PlzA, a c-di-GMP-binding protein, is hypothesized to affect multiple virulence characteristics in *B. burgdorferi*. PlzA has been shown to positively regulate BosR protein levels, which regulates *rpoS* expression. RpoS, in turn, induces expression of genes, such as *ospC*, which are known to be important for mammalian infection as well as for genes involved in chitobiose utilization, which was shown to be important for colonization in the tick. *rpoS* expression is also governed by the Hk2-Rrp2 TCS; thus, PlzA serves as the connector between both TCS in *B. burgdorferi*. There is also a possibility that the PlzA-c-di-GMP complex interacts with another target(s), mediating glycerol utilization, colony morphology, and chemotaxis. The levels of c-di-GMP are controlled by the opposing activities of Rrp1 and PdeA and PdeB, the phosphodiesterases in *B. burgdorferi*. PdeA is an EAL-domain PDE, which hydrolyzes c-di-GMP to pGpG as its major product, which is then subsequently hydrolyzed to GMP by nonspecific PDEs. PdeB contains a HD-GYP domain, which hydrolyzes c-di-GMP into pGpG transiently en route to 2 GMPs as the predominant end product. It is unknown if pGpG plays a role in regulating motility or any cellular function in *B. burgdorferi*. Abbreviations: AcP, acetyl phosphate. Thicker arrow next to PdeA indicates pGpG is the major hydrolysis product; thicker arrow next to PdeB indicates the major product of PdeB hydrolysis is 2 GMPs.

In nature, *B. burgdorferi* cycles between the disparate environments of the *Ixodes* tick vector and mammalian host (Burgdorfer et al., 1982; Levine et al., 1985; Lane et al., 1991; Tsao, 2009; Brisson et al., 2012). During transmission between hosts, *B. burgdorferi* detects changes in its environment, such as pH, CO_2_, nutrient availability, and temperature, and responds appropriately by modulating its gene expression (Carroll et al., 1999, 2001; Revel et al., 2002; Brooks et al., 2003; Ojaimi et al., 2003; Tokarz et al., 2004; Tilly et al., 2008; Samuels and Radolf, 2009; Samuels, 2011). Differential gene expression is essential for the Lyme disease spirochete to endure the diverse and fluctuating environments encountered over the course of the enzootic cycle. Similar to other bacterial species (Stock et al., 2000; West and Stock, 2001; Kazmierczak et al., 2005; Beier and Gross, 2006; Tamayo et al., 2007), *B. burgdorferi* utilizes two-component systems (TCS) to modulate its gene expression (Fraser et al., 1997; Casjens et al., 2000; Radolf et al., 2012; Groshong and Blevins, 2014). Unlike many other species of bacteria, *B. burgdorferi* was reported to encode only two TCS—Hk1-Rrp1 and Hk2-Rrp2—that have demonstrated global gene regulatory capabilities (Yang et al., 2003a; Rogers et al., 2009; Samuels, 2011; Radolf et al., 2012; Groshong and Blevins, 2014). The Hk2-Rrp2 TCS activates the expression of the stationary phase sigma factor RpoS synergistically with RpoN (Burtnick et al., 2007; Ouyang et al., 2008; Blevins et al., 2009), which, in turn, chiefly regulates plasmid-borne genes (Yang et al., 2003a,b; Caimano et al., 2007) and induces the expression of genes, such as *ospC* (Hübner et al., 2001), which are known to be important for mammalian infection (Caimano et al., 2004; Fisher et al., 2005; Caimano et al., 2007; Boardman et al., 2008; Ouyang et al., 2008; Dunham-Ems et al., 2012; Ouyang et al., 2012) as well as genes involved in chitobiose utilization, which has been shown to be important for colonization of the tick (Sze et al., 2013). The Hk1-Rrp1 TCS converges with the Hk2-Rrp2 TCS through the regulator, BosR—a Fur/Per-like transcription factor that has been demonstrated to be essential for expression of *rpoS* (Boylan et al., 2003; Katona et al., 2004; Seshu et al., 2004; Hyde et al., 2009; Ouyang et al., 2009, 2011; Hyde et al., 2010)—which primarily regulates core chromosome-encoded genes (Rogers et al., 2009; He et al., 2011, 2014) and is required for tick colonization (Caimano et al., 2011; He et al., 2011; Kostick et al., 2011). Interestingly, Rrp1, the response regulator, lacks a DNA-binding domain, but instead contains a GGDEF domain, which has been associated with diguanylate cyclase activity (cyclic di-GMP synthase) in *B. burgdorferi* (Ryjenkov et al., 2005). Furthermore, the diguanylate cyclase, Rrp1, is active only when it is phosphorylated, presumably by the histidine sensor kinase, Hk1 (Caimano et al., 2011) (Figure 1). While Hk2-Rrp2 is primarily involved in mammalian host adaptation (Groshong and Blevins, 2014), recent studies suggest that c-di-GMP is a key regulator in the adaptive responses of *B. burgdorferi* to the tick environment (Caimano et al., 2011; He et al., 2011, 2014; Kostick et al., 2011; Pitzer et al., 2011; Sultan et al., 2011). While the genomes of several species of bacteria were reported to encode multiple c-di-GMP-metabolizing enzymes (Galperin et al., 1999, 2001; Galperin, 2004), both bioinformatics and experimental analysis indicate that *B. burgdorferi* possesses only a limited number of genes responsible for regulating c-di-GMP levels—one diguanylate cyclase (*bb0419*/*rrp1*), two distinct phosphodiesterases (*bb0363*/*pdeA* and *bb0374*/*pdeB*), and one c-di-GMP-binding PilZ-domain protein, PlzA (*bb0733*) (Figure 1) (Ryjenkov et al., 2005; Rogers et al., 2009; Freedman et al., 2010; Sultan et al., 2010, 2011; Caimano et al., 2011; He et al., 2011; Kostick et al., 2011; Pitzer et al., 2011).

## The diguanylate cyclase in *B. burgdorferi*

Hk1 (BB0420), the histidine sensor kinase of the Hk1-Rrp1 TCS in *B. burgdorferi*, is comprised of two periplasmic sensor domains (D1 and D2), a conserved cytoplasmic histidine kinase core, REC, and histidine-containing phosphotransfer (Hpt) domains. *hk1* is located on the chromosome next to a response regulatory protein (*rrp1*; *bb0419*) (Fraser et al., 1997), and although it has not been experimentally confirmed that Hk1 is capable of autophosphorylation or that Hk1 is capable of phosphorylating Rrp1, the operon structure of Hk1-Rrp1, as well as genetic analysis, suggests that Hk1-Rrp1 form a TCS (Ryjenkov et al., 2005; Rogers et al., 2009; Caimano et al., 2011; He et al., 2011). Studies by Caimano et al. have shown that while Hk1-deficient spirochetes are able to infect mice normally, they are cleared from the tick within 48 h of the tick feeding. It is hypothesized that the phosphorelay between Hk1 and Rrp1 results in the production of c-di-GMP, which in turn, modulates the downstream gene expression required for survival within feeding ticks (Caimano et al., 2011). Rrp1 is the sole protein in *B. burgdorferi* that harbors a GGDEF domain, and thus, appears to be the only protein capable of producing c-di-GMP. Additionally, the diguanylate cyclase activity of Rrp1 is strictly dependent on the phosphorylation state of the REC domain of Rrp1 (Ryjenkov et al., 2005).

*rrp1* is constitutively expressed *in vivo*, regardless of growth temperature and is increased in the feeding tick relative to the unfed tick (Rogers et al., 2009). Similar to the *hk1* mutant, the *rrp1* mutant was shown to be infectious in mice by needle inoculation, but unable to survive in feeding ticks (He et al., 2011; Kostick et al., 2011). To demonstrate molecular mechanisms underlying the requirement of c-di-GMP for the survival of spirochetes in the fed ticks, microarray analyses of the *rrp1* mutants demonstrated that disruption of c-di-GMP production causes global alteration in gene expression (Rogers et al., 2009; He et al., 2011). Both reports indicated that Rrp1 governs many genes, including the genes required for glycerol transport and metabolism (*glp* genes) (Rogers et al., 2009; He et al., 2011), and mutants defective in glycerol metabolism are attenuated in overall survival in the tick vector (He et al., 2011; Pappas et al., 2011). Interestingly, constitutive expression of the *glp* operon in the *rrp1* mutant only partially rescued survival of the mutant spirochetes in ticks, suggesting that, in addition to *glp*, there are other unidentified factors likely to be critical for the viability of *B. burgdorferi* in its arthropod host (He et al., 2011). The *rrp1* mutant was also reported to exhibit a reduced chemotaxis phenotype and failed to reverse its swimming direction (Kostick et al., 2011). Kostick et al. proposed that this chemotaxis and motility phenotype is likely linked to the fact that the *rrp1* mutant had altered expression of chemotaxis and motility genes (Kostick et al., 2011). Rrp1 was also shown to regulate chitobiose utilization *in vitro* in *B. burgdorferi*. Furthermore, these mutants were unable to transmit to mice via tick bite unless the ticks were supplemented with N-acetylglucosamine (Sze et al., 2013).

The signal that activates Hk1 is not known; however, bioinformatics analysis proposes that Hk1 contains a periplasmic-located sensor domain homologous to the family 3 substrate-binding proteins (SBP_3) (Caimano et al., 2011). SBP_3 family proteins have been shown to bind amino acids or opine molecules (Tam and Saier, 1999). Because of its periplasmic location, the signal is likely to be small enough to permeate the outer membrane of the spirochete and engage the D1 and D2 periplasmic sensor domains of HK1 (Caimano et al., 2011). Based on the phenotypes of the *hk1* mutant, Caimano et al. proposed that the host- and/or tick-derived molecules generated as part of the tick feeding process (either at the bite site or in the tick midgut) may induce the signaling cascade via Hk1, activating the c-di-GMP signal transduction pathway and resulting in the adaptation of *B. burgdorferi* to the callous environment of the tick (Caimano et al., 2011). Although further research is required to establish a definitive biochemical relationship between Hk1 and Rrp1, the similar phenotype exhibited by both the *hk1* mutant and the *rrp1* mutant provides convincing evidence that these two proteins work together to support the production of c-di-GMP. While Rrp2 was thought to be only phosphorylated by its cognate histidine sensor kinase, Hk2 (Burtnick et al., 2007), Xu et al. demonstrated that the high-energy phosphate donor acetyl phosphate (AcP) is also capable of mediating phosphorylation of Rrp2 (Xu et al., 2010). However, this does not appear to be the case for Rrp1 as the phenotype associated with the *hk1* mutant within feeding nymphs suggests that AcP is unable to promote phosphorylation of Rrp1 (Caimano et al., 2011). Together, all of these data suggest that Hk1-Rrp1 is essential for the viability of *B. burgdorferi* in the tick phase of the enzootic cycle, aiding in basic metabolic functions by governing carbohydrate utilization as well as in potential protective functions by shielding the spirochetes from detrimental host factors.

## Phosphodiesterases in *B. burgdorferi*

Once c-di-GMP is synthesized (by Hk1-Rrp1), disposal of the second messenger is critical for modulating the effector protein activity. Degradation of c-di-GMP is achieved by phosphodiesterases, of which there are two domain families: EAL and HD-GYP. EAL-domain-containing PDEs degrade c-di-GMP into 5′-phosphoguanylyl-(3′-5′)-guanosine (pGpG), which is then further degraded by nonspecific cellular PDEs into GMP at a slower rate (Chang et al., 2001; Paul et al., 2004; Christen et al., 2005; Hickman et al., 2005; Schmidt et al., 2005; Tamayo et al., 2005; Schirmer and Jenal, 2009); and HD-GYP-domain PDEs degrade c-di-GMP into pGpG as an transient intermediate en route to producing two GMPs as the major product (Ryan et al., 2006a, 2007, 2009). The amino acid motifs of both proteins (EAL and HD-GYP) are essential for the enzymatic activities of PDEs (Hengge, 2009). In order to reset its c-di-GMP levels, *B. burgdorferi* contains two evolutionary distinct phosphodiesterases: PdeA (BB0363) and PdeB (BB0374) (Sultan et al., 2010, 2011). PdeA contains a functional EAL-domain (Sultan et al., 2010) while PdeB possesses a functional HD-GYP domain (Sultan et al., 2011). Both PDEs were shown to specifically hydrolyze c-di-GMP with high affinity (Sultan et al., 2010, 2011). Furthermore, Sultan et al. measured the phosphodiesterase enzyme activity in the cell lysate of a *pdeApdeB* double mutant, demonstrating that no additional phosphodiesterases are present in *B. burgdorferi*, which is consistent with bioinformatic analysis (Galperin et al., 2001). While both PdeA and PdeB function as phosphodiesterases, the motility and virulence phenotypes of each mutant differs, (Sultan et al., 2010, 2011), suggesting a complex regulatory system that may include differential expression, localization, or regulation of distinct pathways.

Wild-type *B. burgdorferi* cells exhibit the ability to move directionally away from a point of origin in a run-flex/pause-reverse swimming pattern. This movement is decided by the asymmetric rotation of the flagellar motors (Li et al., 2002; Charon et al., 2012). Inactivation of *pdeA* resulted in cells that were unable to reverse their swimming direction (Sultan et al., 2010). The swimming pattern of the *pdeA* mutant is similar to the motility phenotype of the chemotaxis response regulator *cheY3* mutant, which constantly runs and does not flex or reverse (Motaleb et al., 2011). In *Escherichia coli* and *Salmonella enterica*, the CheY protein is a chemotaxis response regulator that binds to the flagellar switch protein, FliM, when phosphorylated, causing the rotation of the flagella to switch from counterclockwise (cells run) to clockwise (cells tumble). Therefore, *cheY* mutants constantly run because they are incapable of switching the rotation of their flagella (Silversmith and Bourret, 1999; Berg, 2003). A motility defect resulting from inactivation of the *B. burgdorferi pdeA* gene is consistent with motility phenotypes reported in similar mutants in other bacteria (Tamayo et al., 2005; Pesavento et al., 2008; Wolfe and Visick, 2008); however, the exact mechanism through which *pdeA* alters motility in *B. burgdorferi* has yet to be determined.

Both *E. coli* and *S. enterica* possess multiple DGCs and PDEs, but only the absence of one specific PDE, YhjH, results in an impairment of motility (Ryjenkov et al., 2006; Girgis et al., 2007; Pesavento et al., 2008). *yhjH* mutants are reported to have elevated c-di-GMP levels, and the inhibition of motility seen in these mutants is dependent on the PilZ protein, YcgR. Thus, when an *ycgR* mutant was constructed in the *yhjH* mutant background, the motility phenotype of the double mutant was reported to be restored back to the wild-type levels (Ryjenkov et al., 2006; Pesavento et al., 2008). YcgR was demonstrated to posttranslationally interact with the flagellar proteins FliG, FliM, or MotA, most strongly in the presence of c-di-GMP, which causes a reduction in the generation of torque and induces a counter-clockwise motor bias (Armitage and Berry, 2010; Boehm et al., 2010; Fang and Gomelsky, 2010; Paul et al., 2010) (see Figure 1 in Armitage and Berry, 2010; and Figure 6 in Fang and Gomelsky, 2010). Thus, the motility defect seen in the *B. burgdorferi pdeA* mutant may be a result of a receptor-c-di-GMP complex interfering with the function of a flagellar protein(s). Furthermore, by constructing a *plzA* mutant in the *pdeA* mutant background, where the cellular c-di-GMP level was 2× higher than that in the wild-type cells, Pitzer et al. demonstrated that PlzA regulates *B. burgdorferi* motility in a different manner from that in *E. coli*, and that elevated c-di-GMP in the *B. burgdorferi pdeA* mutant regulates motility by a mechanism independent of PlzA (Pitzer et al., 2011). Still, it is impossible to ignore the possibility that *B. burgdorferi* may possess another c-di-GMP receptor, which may be responding to the elevated c-di-GMP levels in the *pdeA* mutant and altering its motility. Further studies are needed to confirm these hypotheses.

In contrast to *pdeA*, inactivation of *pdeB* resulted in cells that exhibited a swimming pattern similar to wild-type except they flexed significantly more, suggesting that c-di-GMP may play a role in chemotaxis (Sultan et al., 2011; Kulasekara et al., 2013; Russell et al., 2013). Increased flexing would be expected to be a result of overexpression of the chemotaxis response regulator, CheY3, inhibition of the activity of CheX (the CheY-P phosphatase), or decreased expression of CheX (Motaleb et al., 2005; Pazy et al., 2010; Motaleb et al., 2011). However, Western blot analysis showed that both proteins are expressed at wild-type levels in the *pdeB* mutant, eliminating the possibility of a receptor-c-di-GMP complex directly regulating the expression of chemotaxis proteins (Sultan et al., 2011). However, Sultan et al. demonstrated that a *pdeBplzA* double mutant in *B. burgdorferi* constantly flexed, which is similar to the phenotype of the *cheX* mutant (Motaleb et al., 2005; Sultan et al., 2011), implying that PlzA may control the chemotaxis system either through modulating the activity of CheX or CheY3 (Sultan et al., 2011). Alternatively, the hydrolyzed products of the phosphodiesterases may be responsible for the phenotypic differences observed in the *pdeA* and *pdeB* mutants. A recent study by Stelitano et al. demonstrated that two HD-GYP domain PDEs (PA4108 and PA4781) in *Pseudomonas aeruginosa* are capable of not only hydrolyzing pGpG into two molecules of GMP, but also are capable of using it as a substrate (Stelitano et al., 2013). Moreover, pGpG is reported to be a member of the “nanoRNA” molecules that are known to be involved in regulating gene expression (Goldman et al., 2011; Nickels, 2012; Vvedenskaya et al., 2012; Römling et al., 2013). If this is also true for *B. burgdorferi*, then pGpG may accumulate in the cell under high c-di-GMP concentrations (in the *pdeB* mutant) and potentially exert its role as a signaling molecule; or perhaps the accumulation of pGpG is responsible for some of the phenotypic differences observed in the *pdeA* and *pdeB* mutant.

In addition to their role in regulating motility, PDEs have also been associated in the virulence of some bacteria (Wolfe and Visick, 2008; Römling and Simm, 2009; Ryan et al., 2010). Sultan et al. demonstrated that spirochetes lacking PdeA were unable to infect mice either by needle or tick bite. This deficiency in infection is most likely a result of the inability of the *pdeA* mutant to reverse its swimming direction (Sultan et al., 2010) because inactivation of a chemotaxis histidine kinase, *cheA2*, in *B. burgdorferi* yielded spirochetes that constantly swim in one direction and were unable to reverse and were also unable to establish infection in mice by either needle or tick (Sze et al., 2012). Additionally, intravital microscopy of *B. burgdorferi* in a live mouse indicated that the “back-and-forth” (run-reverse) swimming pattern of wild-type cells is important in transendothelial migration (Moriarty et al., 2008; Norman et al., 2008). However, while spirochetes deficient in PdeA are unable to establish infection in mice, this mutant is capable of colonizing *Ixodes* ticks normally (Sultan et al., 2010). The prospect that c-di-GMP signaling by PdeA is essential for mammalian infection cannot yet be eliminated. Loss of PdeB, on the other hand, had no significant effect in mouse infection by needle inoculation (Sultan et al., 2011), which suggests that PdeA and PdeB may exert their regulatory effects through different mechanisms. Interestingly though, spirochetes lacking PdeB exhibited a survival defect within fed ticks, and ticks infected with PdeB-deficient spirochetes failed to transmit the infection to naïve mice during feeding (Sultan et al., 2011). Independent studies performed by He et al. (2011), Kostick et al. (2011), and Caimano et al. (2011) have shown that *hk1* and *rrp1* are essential for survival of *B. burgdorferi* within fed tick midguts. These results, coupled with other studies, imply that the regulation of c-di-GMP levels by *B. burgdorferi* is central to survival in the tick phase of the enzootic cycle. Further studies are needed to not only elucidate the role c-di-GMP plays in the tick phase of the enzootic cycle, but also specifically the function of PdeA and PdeB during the adaptation process of *B. burgdorferi*.

The significance of *B. burgdorferi* possessing two types of unrelated PDEs in its genome is still unclear, especially considering that HD-GYP PDEs are widespread but not ubiquitous in bacterial genomes (only over 1000 genes have been found among the whole sequenced bacterial genomes) (Römling et al., 2013). The presence of genes encoding for EAL and HD-GYP domain proteins in the *B. burgdorferi* genome suggests that this spirochete regulates c-di-GMP turnover in a sophisticated manner. Indeed, EAL and HD-GYP-domain proteins in other bacterial species have been reported to be associated with known or hypothetical signal input domains that are putatively involved in detecting a wide range of environmental signals (Tal et al., 1998; Galperin et al., 2001; Jenal and Malone, 2006; Ryan et al., 2006b; Barends et al., 2009; Tuckerman et al., 2009). Nevertheless, neither PdeA nor PdeB have been linked to sensory domains; thus, exactly how these PDEs function not only together but also in conjunction with Rrp1 to produce a coherent output signal is still unknown. There is the possibility that *B. burgdorferi* has (at least) two c-di-GMP circuits—one that signals through Hk1-Rrp1-PdeA and one that signals through Hk1-Rrp1-PdeB—which operate in divergent mechanisms in response to environmental signals through two different receptors: the PlzA receptor and an unidentified c-di-GMP receptor. This may mean that the concentrations and activities of PdeA and PdeB would vary over time in response to the fluctuating environmental or cellular conditions. This manner of temporal sequestration was shown in *E. coli*, where the expression patterns of 28 GGDEF/EAL genes were analyzed and most of them exhibited differential expression patterns at different temperatures and growth phases (Sommerfeldt et al., 2009). The c-di-GMP signal transduction systems may also act through the functional sequestration (or compartmentalization) of the c-di-GMP control module components—multiprotein complexes that are comprised of a specific DGC and/or PDE, which are mediated by specific input signals and influence certain effectors and target components (Jenal and Malone, 2006; Christen et al., 2010; Massie et al., 2012). Research has provided increasing evidence that these DGC-PDE interactions do occur. In *Yersinia pestis*, interactions were detected between HmsT (DGC), HmsP (PDE), HmsR (putative glycosyltransferase), and its accessory factor (HmsS)—all of which are attached to the inner membrane and necessary for biofilm-associated phenotypes (Bobrov et al., 2008). Additionally, direct interactions were detected between the HD-GYP-type PDE domain of RpfG and many different GGDEF proteins in *Xanthomonas axonopodi*s (Andrade et al., 2006). With functional sequestration, the close-association of the components may sterically hinder c-di-GMP from diffusing throughout the cell, thus allowing regulation of the localized c-di-GMP concentration in response to specific signals. This may explain the differing virulence and motility phenotypes seen in the *pdeA* and *pdeB* mutants of *B. burgdorferi* (Sultan et al., 2010, 2011). Clearly, further studies are needed to unravel the complicated signaling generated by c-di-GMP-hydrolyzing proteins in *B. burgdorferi*.

## PlzA: the c-di-GMP-binding protein in *B. burgdorferi*

Little knowledge is available concerning the effector mechanisms of c-di-GMP in *B. burgdorferi* or, moreover, any arthropod-borne pathogen. The ability of a second messenger to have numerous effects on cellular behavior lies in the diversity of c-di-GMP receptors. In other species of bacteria, c-di-GMP has been demonstrated to exert its regulatory effects through proteins with cyclic nucleotide monophosphate domains (Tao et al., 2010), ribonucleoprotein complexes (Tuckerman et al., 2011), transcriptional regulators (Hickman and Harwood, 2008; Krasteva et al., 2010; Fazli et al., 2011), GEMM riboswitches (Sudarsan et al., 2008; Smith et al., 2009; Luo et al., 2013), and PilZ domain-containing proteins (Amikam and Galperin, 2006; Ryjenkov et al., 2006; Bian et al., 2013). To date, only one c-di-GMP-binding protein, the PilZ domain-containing protein PlzA, has been identified in *B. burgdorferi* (Freedman et al., 2010; Pitzer et al., 2011). All *Borrelia* species possess PlzA (the PilZ domain-containing protein in the relapsing fever spirochetes is designated as PlzC), and, furthermore, this protein is highly conserved among species of the *B. burgdorferi* sensu lato complex. Interestingly, some *B. garinii* and *B. burgdorferi* isolates possess two PilZ domain-containing proteins—one that is encoded on the chromosome (PlzA) and one that is encoded on linear plasmid 28 (PlzB). Although all three genes encode for a PilZ domain-containing protein, the amino acid identity values of both PlzB and PlzC have diverged enough from PlzA (64% and 65–69%, respectively) to warrant separate gene designations (Freedman et al., 2010). Although PlzA contains the conserved residues of the PilZ domain (RXXXR; DZSXXG; where “X” is any amino acid and “Z” is a hydrophobic residue) (Freedman et al., 2010; Pitzer et al., 2011), it shares poor homology with the PilZ domain-containing proteins of other bacterial species, and it lacks the “PilZ-N” domain (i.e., N-terminal domain of *E. coli* PilZ protein YcgR, which contains a fold similar to the PilZ domain) (Amikam and Galperin, 2006; Ryjenkov et al., 2006). Since PlzA appears to not harbor other identifiable functional domains aside from the PilZ domain, it is considered a “stand-alone” c-di-GMP-binding protein.

The *pilZ* mutants of several bacterial species have been reported to have diverse phenotypes such as altered motility and translucent, rough, dry, and rugose colony morphology (Römling, 2005; Ryjenkov et al., 2006; Lee et al., 2007; Pratt et al., 2007; Yildiz and Visick, 2009; Zorraquino et al., 2013). Unlike the translucent colony morphology of wild-type, the *plzA* mutant of *B. burgdorferi* exhibits opaque colonies, and this phenotype is a direct result of the *plzA* mutation as the morphology is restored back to wild-type upon complementation (Pitzer et al., 2011). The molecular mechanisms underlying the opaque colony phenotype exhibited by the *plzA* mutant have yet to be determined. Many bacteria undergo phase variation between translucent and opaque or smooth and rugose colony morphology. This change is mainly dependent upon variation in the expression of surface polysaccharides (Fries et al., 1999; Yildiz and Schoolnik, 1999; Chang et al., 2009). Previous studies in other bacteria have associated opaque colonies with an increase in capsule production. For example, inactivation of the cyclic adenosine monophosphate (cAMP) receptor protein (CRP) in *Vibrio vulnificus* yielded a mutant that was defective in capsular production and formed translucent colonies compared to the wild-type strain (Kim et al., 2013). Additionally, analysis of opaque and transparent variants of a *Streptococcus pneumoniae* clinical serotype revealed that opaque variants produced an extracellular matrix, had 100-fold greater *in vitro* adherence, and showed increased virulence *in vivo* compared to the transparent bacteria (Trappetti et al., 2011). Furthermore, the PelD protein of *P. aeruginosa* was shown to be a c-di-GMP receptor which mediates the regulation of Pel polysaccharide—an extracellular adhesion necessary for the formation and maintenance of biofilms—in a c-di-GMP-dependent manner (Lee et al., 2007). Thus, one possibility is that the *plzA* mutant spirochete has increased levels of surface polysaccharides. However, the type of polysaccharide that has increased expression in the mutant, or even if it is a polysaccharide (and not another type of adhesion or surface molecule) is unknown. Moreover, if and how *plzA* regulates polysaccharide production or if they play a role in *B. burgdorferi* pathogenesis is also currently unknown. Clearly, there are more questions than answers concerning polysaccharide production, indicating the need for further research in this area.

While the swimming pattern (run-flex/pause-reverse) of the *plzA* mutant was indistinguishable from its parental wild-type strain, the swarming motility of these mutant cells were attenuated compared to wild-type cells or the isogenic complemented strain (Pitzer et al., 2011). PilZ proteins were reported to control motility in response to the concentration of c-di-GMP in other bacterial species. For example, the c-di-GMP-binding PilZ protein, DgrA, in *Caulobacter crescentus* was shown to diminish synthesis of FliL when complexed to c-di-GMP, resulting in an alteration of motility (Christen et al., 2007). This scenario is unlikely to occur in the *plzA* mutant of *B. burgdorferi*, however, because the levels of major motility and chemotaxis proteins (i.e., FliL, FliG1, FliG2, FliM, MotB, FlaB, and CheY3) were shown not to be altered (Pitzer et al., 2011). Furthermore, PlzA lacks the N-terminal YcgR domain (Freedman et al., 2010; Pitzer et al., 2011), which has been reported to be important to interact with the flagellar switch proteins to control bacterial motility (Fang and Gomelsky, 2010). As discussed earlier, *B. burgdorferi* may possess an additional c-di-GMP-binding protein(s), which controls motility in response to c-di-GMP levels in a mode similar to that reported in *E. coli* or *C. crescentus*. Studies have shown that PilZ-domain proteins that possess two domains (e.g., PilZ and YcgR-N) undergo a conformational change upon binding to c-di-GMP that exposes a new interaction surface. For example, binding of c-di-GMP to the *Vibrio cholera* PilZ-domain protein, VCA0042, induces a conformational change in the loop connecting the C-terminal PilZ domain and the N-terminal YcgR-N domain, bringing the two domains in close proximity with c-di-GMP at the mutual interface to form a new allosteric interaction surface (Benach et al., 2007). Additionally, unlike VCA0042, PP4397 from *Pseudomonas putida* binds two molecules of c-di-GMP in its YcgR-N and PilZ domain junction, which results in a change of its quaternary structure from a dimer to a monomer (Ko et al., 2010). These studies suggest that different PilZ-domain proteins display diverse binding stoichiometries and mechanistic interactions. One could hypothesize that in *B. burgdorferi* PlzA undergoes a similar conformational change upon binding to c-di-GMP, and the generation of this new molecular surface constitutes the readout of this small signaling protein by providing a highly-charged interaction surface for high-affinity regulatory interactions with downstream target proteins. In support of this, the solution structure of the *P. aeruginosa* single-domain PilZ protein, PA4608, in complex with c-di-GMP, was recently solved by NMR spectroscopy (Habazettl et al., 2011). PA4608 is a stand-alone PilZ-domain protein that was shown to undergo conformational changes at both termini of the protein while maintaining a certain degree of flexibility upon binding to c-di-GMP, which resulted in a severe rearrangement of surface charges. The rearranged termini expose a highly negatively-charged surface on one side of the complex to a potential effector protein (Habazettl et al., 2011). Thus, even though PlzA is a stand-alone PilZ-domain protein and lacks the N-terminal YcgR domain (Freedman et al., 2010; Pitzer et al., 2011), it is still plausible that it functions as a c-di-GMP effector protein. If PlzA is the only c-di-GMP-receptor protein in *B. burgdorferi* and it does undergo a conformational change similar to PA4608, then the degree of flexibility maintained in the complex may influence the target specificity (the downstream target) of PlzA, especially if different c-di-GMP circuits are separated spatiotemporally through compartmentalization. Moreover, the target specificity of PlzA may also be influenced by several other potential factors (e.g., c-di-GMP levels, other proteins, co-factors, temperature, etc.), which may directly be indicative of the local environment. Further studies are needed to demonstrate this in addition to the downstream targets of PlzA.

*plzA* has been shown to be constitutively expressed during *in vitro* culture (Freedman et al., 2010)—similar to previously published microarray data (Ojaimi et al., 2002; Revel et al., 2002; Brooks et al., 2003)—and this expression is not dependent on temperature (Freedman et al., 2010). Transcripts of *plzA* were also detected in murine bladders as late as 13 weeks after infection (which was the last time point analyzed), indicating that expression of *plzA* is maintained throughout the enzootic cycle (Freedman et al., 2010). Furthermore, the *plzA* mutant is significantly less infectious in mice than the wild-type parental strain (Pitzer et al., 2011). This observed decrease in virulence is consistent with mutations in the PilZ proteins of other bacteria. While motility may play a role in the reduced virulence of the *B. burgdorferi plzA* mutant, as wild-type motility is critical for infectivity, future studies are needed in order to precisely delineate this mechanism. Interestingly, expression of *plzA* was reported to be significantly increased in ticks after a bloodmeal (Freedman et al., 2010), and *plzA* mutant cells were shown to be comprised in fed ticks (Pitzer et al., 2011). While the mechanism resulting in this survival defect in fed ticks is unknown, it is most likely that PlzA (and not solely motility) is responsible for the survivability in the fed tick because other mutants defective in motility (e.g., *cheA2*, *pdeA*) are capable of not only surviving, but multiplying before and after a blood meal (Sultan et al., 2010; Sze et al., 2012).

He et al. have recently shown that PlzA-c-di-GMP functions in linking the two global TCS in *B. burgdorferi*—Hk1-Rrp1 and Hk2-Rrp2 (He et al., 2014). Furthermore, He et al. also demonstrated that PlzA modulates expression of *rpoS* through BosR and this occurs in a mechanism independent of Rrp1 (He et al., 2014) (Figure 1). This suggests that aside from functioning as a c-di-GMP-binding protein, PlzA also has other roles in *B. burgdorferi*. Thus, it is likely that PlzA either plays a direct role in the survivability of *B. burgdorferi* in fed ticks or regulates a virulence determinant(s), which influences survival in the tick as well as infectivity in the mouse (He et al., 2014). Furthermore, it is also possible that the opaque colony morphology of the *plzA* mutant reflects an alteration in a cell surface membrane structure, which may be related to both the decreased survivability of the mutant cells in the tick and attenuation in the mouse infection model (Pitzer et al., 2011). Recent studies have demonstrated that *glp* genes are important for the survival of *B. burgdorferi* in the tick (Pappas et al., 2011) and are regulated by Rrp1 (Rogers et al., 2009; He et al., 2011). If PlzA plays an important role in mediating responses initiated by the Hk1-Rrp1, it is probable that the defect seen in the *plzA* mutant is related to a defect in glycerol utilization, and constitutive expression of the *glp* genes in the *plzA* mutant would be expected to rescue the survival defect of the mutant in the tick. However, constitutive expression of the *glp* operon in the *rrp1* mutant only partially rescued the survival of the mutant in the tick (He et al., 2011), and as such, it would be expected the same would occur in the *plzA* mutant, especially if PlzA controls other cellular processes that may be important in virulence and/or survival in both hosts in a c-di-GMP-dependent or -independent manner (He et al., 2014). Moreover, it is unknown if tick-derived elements, host-derived products acquired from the tick's blood meal, or salivary gland factors were at fault for the reduced survival of the *plzA* mutant in the tick (Sonenshine et al., 2002; Sonenshine and Hynes, 2008; Hajdušek et al., 2013). Additionally, the mechanism resulting in the decreased infectivity of the *plzA* mutant in the mouse also has yet to be elucidated. It is known that *B. burgdorferi* utilizes different means to survive in its disparate hosts, but the role PlzA plays in that survival has yet to be revealed. Current research has just begun to scratch the surface, and future studies are needed to unravel the extensive effects both PlzA and c-di-GMP are anticipated to have on *B. burgdorferi*.

## Conclusions

Although 25 years has passed since the discovery of c-di-GMP in bacteria (Ross et al., 1987; Römling et al., 2013), the study of c-di-GMP signaling in *B. burgdorferi* has just begun and is still in its infancy. A great deal more research is needed in order to provide a complete inventory of the effectors molecules and targeted processes of this second messenger. While modulation of c-di-GMP represents an attractive target for controlling the progression of disease, the molecular and biological effects of this signaling system within *B. burgdorferi* must be understood before potential mechanisms of interference can be proposed. Thus, understanding the c-di-GMP signaling cascade of *B. burgdorferi* will prove invaluable to learning how to therapeutically exploit this second messenger system in the treatment and/or prevention of Lyme disease.

### Conflict of interest statement

The authors declare that the research was conducted in the absence of any commercial or financial relationships that could be construed as a potential conflict of interest.
